# Curcumin: New Insights into an Ancient Ingredient against Cancer

**DOI:** 10.3390/ijms20081808

**Published:** 2019-04-12

**Authors:** Ella Willenbacher, Shah Zeb Khan, Sara Cecilia Altuna Mujica, Dario Trapani, Sadaqat Hussain, Dominik Wolf, Wolfgang Willenbacher, Gilbert Spizzo, Andreas Seeber

**Affiliations:** 1 Department of Internal Medicine V: Hematology and Oncology, Medical University of Innsbruck, Innsbruck 6020, Austria; ella.willenbacher@i-med.ac.at (E.W.); dominik.wolf@i-med.ac.at (D.W.); wolfgang.willenbacher@tirol-kliniken.at (W.W.); gilbert.spizzo@i-med.ac.at (G.S.); 2 Department of Clinical Oncology, BINOR Cancer Hospital, Bannu 28100, Pakistan; skhanizhere0@gmail.com; 3 Department of Molecular Biology, Laboratorio Blau, Caracas 1071, Venezuela; altunamujica.md@gmail.com; 4 Department of Oncology and Hematology, University of Milan, European Institute of Oncology, 20122 Milan, Italy; dario.trapani@ieo.it; 5 Medical Oncology Department, KAMC NGHA, Riyadh 14413, Saudi Arabia; oncologysh@gmail.com; 6 Oncotyrol, Center for Personalized Cancer Therapy, Innsbruck 6020, Austria; 7 Oncologic Day Hospital, 39042 Bressanone, Italy

**Keywords:** curcumin, complementary medicine, cancer treatment, supportive care, antioxidants, anti-inflamation

## Abstract

Cancer patients frequently use complementary medicine. Curcumin (CUR) and its derivates (from the extract of *Curcuma longa* L.) represent some of the most frequently used ones, having a long history in traditional Asian medicine. CUR was demonstrated, both in vitro and in vivo, to have significant anti-inflammatory effects, thus potentially counteracting cancer-promoting inflammation, which is a hallmark of cancer. CUR modulate a plethora of signaling pathways in cancer cells, comprising the NF-κB (nuclear factor k-light-chain-enhancer of activated B cells), the JAK/STAT (Janus-Kinase/Signal Transducers and Activators of Transcription), and the TGF-β (transforming growth factor-β) pathways. Furthermore, CUR confers properties of electron receptors, which destabilize radical oxygen species (ROS), explaining its antioxidant and anti-apopototic effects. Although CUR has a low bioavailability, its role in advanced cancer treatment and supportive care was addressed in numerous clinical trials. After promising results in phase I–II trials, multiple phase III trials in different indications are currently under way to test for direct anti-cancer effects. In addition, CUR exerts beneficial effects on cancer treatment-related neurotoxcity, cardiotoxicity, nephrotoxicity, hemato-toxicity, and others. More efficient galenic formulations are tested to optimze CUR’s usability in cancer treatment. This review should provide a comprehensive overview of basic science, and pre-clinical and clinical data on CUR in the field of oncology.

## 1. Introduction

Cancer patients frequently use natural and herbal products during cancer treatment. A recent Italian review reported that half of all cancer patients use complementary and/or alternative medical (CAM) approaches [1]; however, the choice of CAM varies widely and may also depend on the cultural setting and availability of interventions and drugs. 

Oncologic healthcare professionals should provide non-judgmental and evidence-based support to cancer patients and guarantee the safety of cancer treatment by adopting a clinical and research-based approach to complementary and alternative medicine. Numerous natural products or substances derived from plants or other life forms were evaluated in different medical conditions, especially in cancer. 

Curcumin (CUR) and its derivates represent one of these products and are derived from the extract of *Curcuma longa* L. (turmeric rhizomes) [1,2]. Turmeric is a plant used for thousands of years in Asia, especially in the Vedic culture in India, where it is frequently used as a culinary spice or a dye and represents a component of traditional Chinese medicine and other medical cultures [3]. Curcuminoids include also demethoxycurcumin and bisdemethoxycurcumin, and most preparations that are available today are heterogenic biological mixtures of extracts of *Curcuma longa*. One must take into account that curcuminoids are of variable solubility [4], and the major problem when using curcuminoids is represented by its low bioavailability because of poor solubility [5]. Hence, high single doses of CUR are required to achieve detectable levels in serum of healthy volunteers [6]. Thus, different strategies were tested to overcome these limits, such as liposome-based formulations, and emulsion or microsphere preparations of CUR [7,8,9], all of which were developed with the ultimate goal of optimizing its bioavailability. 

However, despite these unfavourable galenic properties, CUR displays several positive effects in vivo and in vitro. Due to its high concentration in the gastrointestinal tract for example, Shen et al. demonstrated a regulative effect of CUR with respect to microbial composition in the gastro-intestinal (GI) tract of C57BL/6 mice [10]. One of the most promising CUR effects, however, appears to be its anti-inflammatory potential. Tabrizi et al. summarized these effects by showing that CUR modulates inflammatory biomarkers such as IL-6 (Interleukin-6) and hs-CRP (high-sensitivity c-reactive protein) [11]. In line with these observations, a Cochrane analysis showed that CUR might be a safe and an effective therapy for maintenance of remission in quiescent ulcerative colitis [12].

Chronic inflammation is characterized as an emergent “hallmark of cancer” by driving malignant transformation on cancer progression [13]. Therefore, the capacity of the proven anti-inflammatory compound CUR to modulate signaling pathways in cancer cells was widely investigated. The most important of these are the NF-κB (nuclear factor k-light-chain-enhancer of activated B cells) pathway [14], the JAK/STAT (Janus-Kinase / Signal Transducers and Activators of Transcription) pathway [15], and the TGF-β axis [16]. All of these were demonstrated to be potentially modulated by CUR [14,15,16]. 

These pathways are particularly important in multiple neoplasia, and one of the most interesting aspects of recent research on CUR is the focus on cancer treatment and prophylaxis. Given the complexity of cancer medicine, natural products such as CUR might play a role in specific treatments, as well as in in supportive care. Thus, based on these observations, the present review aims to provide an overview on the most important aspects of CUR and cancer, focusing both on potential mechanisms of action, as well as results of clinical trials.

## 2. Effects of Curcumin on Tumor Cells, Metabolism, and Signaling Pathways

In addition to its anti-inflammatory properties, CUR was shown to exihibit antioxidant effects in cellular models both in vitro and in vivo. A molecular structure rich in phenol groups and biophysical characteristics allow CUR to interact with many different proteins at different stages, which may explain the diverse antitumor effects [17]. The regulation of enzymes and the activation and deactivation of growth pathways and programmed cell death make CUR a potential therapeutic agent for a broad spectrum of cancers, since the effects observed in cancer cell models could not be replicated in non-neoplastic cells. This fact may explain the low toxicity reported in interventional trials in humans [18]. 

It is still to be determined if the resulting putative anti-cancer effects derive from the products available after degradation, since CUR has a low oral bioavailability, undergoes first-pass metabolism, is hydrophobic, and consecutively does not reach high plasma levels [19]. Therefore, the synthesis of curcuminoid analogs may improve the bioavailability and effectiveness of CUR [20]. 

The presence of phenolic analogs in CUR confers properties of electron receptors, which destabilize radical oxygen species (ROS), explaining the observed antioxidant effects. There are multiple in vitro models demonstrating the avidity for electrons and an ROS scavenging activity [21]. CUR is, therefore, active in the repair mechanismns of DNA due to ultraviolet (UV) damage and stress, and it reduces ROS compounds that play a role in early carcinogenesis [22]. 

CUR further influences cytochrome P450 isoforms and has a direct effect on phase I and phase II metabolism, inhibiting the production of toxins that potentially act as carcinogens. This action on early cancer-initiating events may at least in part explain the protective potential of CUR with respect to malignant transformation and cancer progression [23]. 

In tumor cells, the interaction with ROS is considered as one of the main triggers of apoptosis. Syng-ai et al. [23] demonstrated that depletion of gluthatione sensitized cells to CUR effects, and also downregulated the expression of Bcl-2 (B-cell lymphoma 2) in breast cancer and hepatoma cell cultures, which may be responsible for making them more vulnerable to apoptotic death. These effects were not observed in normal cells, which did not experience variation in superoxide generation. 

The modulation of the inducible nitric oxide synthase gene expression derives lower concentrations of nitric oxide in macrophages, resulting in inhibition of carcinogenesis [24]. The effects of curcumin on GST (Glutathione-S-Transferase) metabolism, paired with the inhibition of immortalizing pathways and other enzymes that result in free ROS, contribute in the preliminary stages of cancer formation, and have direct involvement in the induction of apoptosis in tumor cells. 

The interaction with other proteins has an anti-inflammatory effect. Pignanelli et al. [20] synthesized CUR analogs capable of efficiently killing triple-negative, inflammatory breast, p53-negative colorectal, and different blood cancer cell lines, by manipulating and increasing ROS species specifically in these cells, which translates to the induction of apoptosis. These analogs also proved to be more toxic and effective than natural CUR, with lower intracellular concentrations achieving the same effects.

CUR also has a direct effect on the synthesis of pro-inflammatory cytokines that perpetuate inflammation in favor of tumor growth. The inhibition of the COX2 (cyclooxygenase-2) and NF-κB genes derives an anti-inflammatory effect with a reduction in the synthesis of cytokines and pro-mitotic proteins, since genes regulated by NF-κB include cyclin-D1, Bcl-2, MMP-9 (matrix metalloproteinase-9), and several cytokines such as TNF-α (tumor necrosis factor-α) and many others [25]. In rat models of hemorrhagic resuscitation, Maheshwari et al. demonstrated that the exposure to CUR resulted in significant reduction of of pro-inflammatory cytokines such as IL-1α, IL-1β, IL-2, IL-6, and IL-10 to almost normal levels [26].

The antitumor effects of CUR revolve around the induction of apoptosis through the complex interaction of proteins in the STAT-3, HIF1/ROS (hypoxia inducible factor 1/reactive oxygen species), Wnt/β-catenin, and Sp-1 (specificity protein 1) pathways, as well as induction of the caspase pathways, mainly through the activation of caspase-3 and caspase-8, and endoplasmatic reticulum and mitochondrial stress [27]. The downregulation of the expression of anti-apoptotic genes such as Bcl-2 and Bcl-X makes cancer cells more vulnerable to apoptosis and, in cellular models with Bcl-2 overexpression, some analogs deactivate the Fas (CD95)-associated protein with death domain, resulting in programmed cell death [28]. CUR can also inhibit growth promoters and growth factors, such as EGFR (epithelial growth factor receptor) and cyclin D1 [28]. 

In prostate cancer cell and xenograft murine models, cyclohexanone curcumin analogs decreased invasion, migration, and ability to metastasize due to decreased matrix metalloproteinase production [29]. Other observations potentially explaining anti-metastatic properties include modulation of vascular endothelial growth factor (VEGF) synthesis, thus directly impacting angiogenesis [26]. Kunnumakkara et al. reported inhibition of VEGF production in orthotopic pancreatic and ovarian cancer cells implanted in mice [30].

A third path to cell death, autophagy, also known as type II cell death, is linked to CUR activity. The activation of the mTOR (mechanistic target of rapamycin) pathway seems to regulate autophagy in cancer cells through the complex mTORC1, and CUR deactivates this regulation by deactivation of the PI3K (Phosphoinositide 3-kinase)/Akt/mTOR pathway, extensively demonstrated in multiple cell culture models [31]. 

Another interaction with the mTOR pathway that leads to cell death is the inhibition of the aerobic glycolysis in anaerobic conditions (Warburg effect), which is promoted in cancer cells by the HIF1α pathway. Through the direct downregulation of pyruvate kinase M2 and modulation of mTOR, CUR decreases intracellular levels of HIF1α and glucose uptake, contributing to apoptosis [32].

The hepatocyte growth factor (HGF)/mesenchymal–epithelial transition factor (MET) axis is a further disregulated pathway in cancer which provokes tumor proliferation, as well as therapy escape [33]. As such, it is considered as a poor prognostic marker. In vitro data using different lung cancer cell lines showed that CUR inhibits HGF-induced migration and blocks the c-Met/Akt/mTOR signaling pathway. In a further mouse model, the authors illustrated that CUR induces an upregulation of epidermal markers (i.e., E-cadherin) and a downregulation of mesenchymal factors (i.e., vimentin). The authors concluded that these results suggest that CUR could inhibit epidermal-to-mesenchymal transition (EMT) by targeting the HFG/MET pathway [34]. 

CUR also demonstrates multiple effects on cancer stem cells, which remain dormant and unaffected by traditional chemotherapies and, therefore, enable tumor re-growth, leading to overt relapse after treatment. Varying doses and duration of administration of CUR on cancer stem cells using a wide array of cell cultures models (breast, colon, and lung cancer) showed antitumor activity, with a reduction in the formation of new spheres, activation of caspases, and other pro-apoptotic proteins, as well as downregulation of markers for stemness (CD133, CD44, ALDH1 [aldehyde dehydrogenasis]), increased availability, and activation of PARP (poly[ADP-ribose]-polymerase) inducing sensitivity to anti-cancer drugs like 5-fluoruracil and dasatinib in colon cancer cells, and cisplatinum in lung cancer cells [35]. Hedgehog signaling (HH) is a substantial pathway in cancer stem cells and in carcinogenesis. In the liver for example, HH signaling activation leads to the development of cirrhosis and liver cancer. The activation of this pathway correlates with a higly aggressive cancer by enhancing development of metatastasis and tumor growth [36]. HH signaling is essential to maintain the stemness in of cancer stem cells. In various two- and three-dimensional (2D and 3D) culture models, CUR decreased the activity of cancer stem cells by inhibiting tumor cell proliferation, downregulating cancer stem-cell markers (i.e., CD44 and Oct4 [octamer-binding transcription factor 4]), and inducing apoptosis [37,38,39]. Another research group showed in an in vitro experiment that CUR could not only inhibit proliferation and invasion by inhibiting the HH pathway, but could also induce expression of EMT-related markers [40].

There is evidence for the existence of synergistic effects with traditional anti-cancer therapies, due to the induction of apoptosis through the targeting of several pro-apoptotic and anti-proliferative pathways in different in vitro models. In an experimental model published by Zhang et al., the combination of CUR with cisplatinum in lung cancer xenograft murine models effectively demonstrated reduction of Sp-1 binding to CTR1 (copper uptake protein 1) promoters, increasing expression of CTR1 and Sp-1 and, thus, the uptake and sensitivity to cisplatinum in these cells [35]. Interestingly, antitumor effects were increased when both cisplatinum and CUR were combined and administered in increasing dosages, achieving significantly higher cell death and tumor regression rates compared to their application as monotherapy. Another possible synergistic interaction is with anti-EGFR therapy, targeting the EGFR/MAPK (mitogen-activated protein kinase) pathway known to stimulate cell proliferation [41]. Chen et al. [42] observed that simultaneous treatment with the anti-EGFR antibody cetuximab and CUR demonstrated suppression of the EGFR/MAPK pathway in oral cancer cells resistant to platinum. 

Multiple observations of curcumin’s anti-cancer effects were made in colon cancer xenograft models [43]. James and collegues demonstrated a direct effect on colon cancer stem cells derived from patients’ metastatic tissue, noting a more dramatic decrease in formation of spheroids and expression of stemness biomarkers in cultures that received a combination of FOLFOX and CUR, suggesting there might be a synergistic effect, expressed also in longer DFS (disease-free survival) intervals of patients that received experimental therapy in this trial [44]. A summary of the interactions of CUR is listed in Table 1.

## 3. Clinical Data of Curcumin as a Therapeutical Anti-Cancer Compound

The first monotherapy assessment of pharmacokinetics and activity of CUR was reported in a phase I clinical trial assessing the safety of CUR in colorectal cancer patients, in a dose-finding design of an oral formulation [45]. No dose-limiting toxicities were observed in the 15 patients enrolled. Two patients (13%) exhibited stable disease by radiologic criteria after two months of treatment, receiving the treatment for a total of four months. One patient taking 450 mg of CUR daily and one patient allocated in the full dose group (3600 mg of CUR daily) developed significant diarrhea. However, the patient in the lower-dose cohort was able to optimize the management of this side effect with loperamide, while the patient taking the highest dose discontinued treatment, with rapid improvement of side effects. Accordingly, the authors defined a recommended phase II dose of 3600 mg daily as being suitable for further evaluations. 

In a phase I dose-finding trial, CUR was analyzed in combination with docetaxel 100 mg/m^2^ in 14 breast cancer patients [46]. Among eight patients evaluable for response, five (63%) showed a partial response. The maximal tolerated dose was established at 6000 mg daily for one week followed by two weeks off. 

In a phase II clinical study conducted in patients with advanced pancreatic cancer, patients were enrolled to receive 8000 mg of CUR daily p.o. (per os) in combination with gemcitabine 1000 mg/m^2^ intravenously weekly for three of four weeks [47]. CUR was split into two daily doses. Nearly one-third of the patients (*n* = 5) discontinued CUR due to toxicity and continued gemcitabine monotherapy. The principal adverse event causing the discontinuation of CUR was upper abdominal pain, presenting on average within two weeks from the beginning of the treatment, and not ameliorating with reduction of the CUR dose. Indeed, patients stopping CUR achieved a complete reversal of the symptoms, with no residual impairments. Time to progression was 2.5 months and overall survival was five months, consistent with the benefit achieved by gemcitabine in monotherapy of historical controls. In terms of disease control, five of 11 patients evaluable for response (45.5%) showed a clinical benefit, of which one (9.1%) had a partial response and four (36.4%) had a stable disease. The authors concluded that high-dose oral CUR in combination with chemotherapy is not an effective strategy, as the trial failed to demonstrate a safe feasibility of the combination regimen. 

An additional phase II clinical trial conducted in patients with pretreated advanced pancreatic cancer (*n* = 25) receiveed oral CUR 8000 mg daily as monotherapy until disease progression [1]. Of the patients evaluable for response (*n* = 24), two patients (8.3%) showed a clinical response. As expected, only low levels of CUR were detectable in plasma, i.e., 22–41 ng/mL at steady state. However, some pharmacodynamic assays along with the radiological tumor responses suggested a biologic activity at low bio-disponible plasma concentrations, with effects exerted on the expression of COX-2, NF-κB, and pSTAT3; no correlation of the cytokine change was demonstrated with either biologic activity or with clinical benefit [48]. 

The combination of CUR with tyrosine kinase inhibitors was investigated [49] in a cohort of patients receiving imatinib for CML (chronic myeloid leukemia) (*n* = 50), with or without turmeric powder (1500 mg daily). Patients who received CUR and imatinib together achieved a higher rate of clinical complete response compared to imatinib monotherapy. However, this finding was not statistically significant. Another imatinib combination was tested in a single patient with a pre-treated metastatic adenoid cystic parotid tumor, harbouring a c-KIT mutation [50]. The formulation used was intravenous CUR 225 mg/m^2^ twice a week plus oral CUR 168 mg daily. The patient achieved a partial response still ongoing after 24 months of treatment. 

As proof of concept, Capalbo et al. [51] provided a pivotal experience of a combination of CUR with monoclonal antibodies. The report described the case of an elderly platinum pre-treated cutaneous squamous cell carcinoma patient, receiving the EGFR monoclonal antibody blocker cetuximab combined with daily oral CUR phospholipid supplement (500 mg). Partial response was described, persistent for 11 months, with no evidence of tumor progression at the time of the last follow-up. The authors justified the decision to combine cetuximab with CUR as supposing to optimize the control of EGFR blocker-related skin adverse events based on a report (*n* = 52) by Wada et al. [52] and possibly potentiate the antineoplastic activity, overcoming the emergent resistance to EGFR blockers [53]. 

As a further attempt to optimize CUR bio-availability through drug delivery, liposomal CUR was tested in a phase I clinical trial in patients with advanced pre-treated solid tumors (*n* = 32) [54]. The liposomal formulation was administered intravenously as weekly infusion for eight weeks. Two patients experienced grade-3 anaemia and one patient experienced grade-3 hemolysis. One patient showed a clinical benefit, with a stable disease after four weeks. 

The role of CUR was investigated in the prevention of cancer and treatment of pre-invasive tumors, reporting some preliminary results [55,56,57]. Oral and topic formulations were proposed, tailoring high-risk patients with CUR-based pharmacological interventions and tracking the reduction of pre-invasive lesions when exposed to CUR and related compounds. However, no definite role was defined in these settings, with discordant results in the clinical series, and results of ongoing trials are awaited. 

The role of CUR in cancer treatment was addressed by numerous trials. Although apparently working at low plasma concentration, systemic bio-availability still represents an issue when dealing with CUR experiments in humans. Indeed, the optimization of CUR bio-availability through drug-delivery strategies provided more significant results, suggesting that in vitro and in vivo antitumoral activity can be replicated in the clinical setting by different pharmacological strategies. However, some of the results provided so far are no more than proof-of-concept studies, particularly for the combination with targeted agents, where only a few positive experiences are reported. Moreover, no phase III clinical trial provided results on the antitumor efficacy compared to the standard treatments, with efforts warranted in the definition of the disease most likely to benefit along with predictive biomarkers for better patient selection. For this, despite promising results and early signals of benefit, at present, no specific recommendation should be provided for CUR in the treatment of cancer, as data of safety are restricted to monotherapy and few combinations with selected agents, and data of activity are not mature enough. Eventually, the use of CUR to “adjuvate” the standard treatments should be discouraged unless new results will identify a precise setting of care as a pharmacological compound. 

## 4. Role of Curcumin in Reducing Side Effects of Cytotoxic Drugs

Myelosuppresion is the most common side effect associated with chemotherapy. A murine model showed that, when CUR was administered after carboplatinum, it was able to reduce the length and depth of myelosuppression via reducing the DNA damage in the bone marrow [58]. 

One study found that use of CUR along with doxorubicin remarkedly reduced the myocardial damage in albino rats [59]. The cardiac damage markers such as LDH (lactat dehydrogenasis) and CPK (creatininekinase) were also reduced, suggesting a protective role to anthracycline-induced cardiotoxicity. Another animal study on rats showed that cardiac damage due to doxorubicin was reduced due to CUR use [60]. CUR was shown to reduce the grade of lipid peroxidation and glutathione depletion—markers of oxidative stress. Furthermore, the levels of troponin, LDH, and a cardiac isoform of LDH decreased, along with an attenuation of pro-apoptotic signaling in myocardial cells, including pro-inflammatory mediators.

One experimental study on rats reported that CUR is effective in reducing weight loss and intestinal mucosal damage in 5-flurouracil use [61]. Importantly, no loss of the antineoplastic benefit of 5-flurouracil could be observed. 

In a phase I study, a mucoadhesive formulation containing, among other compounds, CUR was tested as a mouth-washing solution for head and neck cancer patients. No genotoxic cellular damage was observed [62]. The formulation was shown to be clinically active with no apparent systemic adverse events. 

The liver is the main site of metabolism of most chemotherapeutic drugs, and liver damage is a common toxicity of many cancer medicines. Cisplatinum damages the hepatocytes, resulting in the increase of serum ALT (alanine aminotransferasis) and AST (aspartate aminotransferasis). CUR pretreatment improved the hepatocyte damage due to cisplatinum in rats [63]. Another investigation showed that histopathologically confirmed methotrexate-induced liver damage in albino rats can be markedly reduced by post-methotrexate CUR administration [64].

Mitomycin-C is known for potentially causing severe toxicites to both kidney and bone marrow. An investigation was performed using a breast cancer xenograft model in which CUR was administered together with Mitomycin-C [65]. It showed that CUR decreased the kidney damage induced by Mitomycin-C, while at the same time sensitizing tumor cells to Mitomycin-C. Cisplatinum is broadly recognized to be able to provoke severe kidney injury causing acute kidney damage. When CUR was administered to the rats for three consecutive days concomitantly with cisplatinum, CUR showed a renal protective effect preserving kidney function by preventing mitochondrial bioenergetic alterations [66]. 

All the above evidence about CUR protective effects on chemotherapy-induced toxicities mostly are derived from animal experimental or in vitro studies. There is an unmet need to perform clinical trials in the future to confirm safety and efficacy of CUR in humans undergoing cancer therapy. As CUR is an unstable product with poor absorption and rapid systemic elimination, formulations with better bioavailability should be the focus in these these future investigations [67].

## 5. Perspective

Various preclinical studies highlighted the effects of CUR on NF-kB, STAT3, COX2, and CXCL-1 (chemokine [C-X-C motif] ligand 1) activity, leading to reduced inflammation and eventually also impacting tumor progression. Despite these promising in vitro and in vivo results, a clear clinical benefit could not be demonstrated in clinical trials. Thus, currently, a large amount of larger clinical studies are ongoing to investigate the effect of CUR in cancer patients in more detail. 

At the moment, CUR is under evaluation against placebo in a phase II clinical trial (NCT02944578) in HIV (human immunodeficiency virus) -infected women with high-grade squamous intraepithelial lesions of the cervix. In another study, oral CUR is being tested for the treatment of cervical intraepithelial neoplasia (NCT02554344), assessing drug-induced tumor regression. CUR is also proposed as a chemo-preventive agent alternative to other anti-inflammatory drugs, as it inhibits COX activity and is, therefore, being tested in patients with familial adenomatous polyposis (FAP) (NCT00927485). For high-risk men under active surveillance for a biopsy-proven low-risk prostate cancer, a bioavailable formulation of CUR is expected to control disease and reduce the rates of clinically indicated interventions for prostate cancer, including the progression of locoregional disease or spread to distant sites requiring surgery or hormone therapy, respectively (NCT03769766). Similarly, the NCT01975363 trial is assessing the role of daily oral CUR for obese women, as a risk-reducing strategy for high-risk patients defined by either genetic risk, namely harboring *BRCA* mutations, or clinically, when diagnosed with in situ ductal carcinoma. 

In colorectal cancer, CUR showed its role in FAP and is now being tested actively in combination with conventional chemotherapy either to overcome resistance or to enhance the chemotherapeutic effects. In particular, an early study is testing CUR and a phospholipid mix with 5-floururacil, assessing the feasibility, safety, and effectiveness of the combination strategy (NCT02724202; NCT02439385). Moreover, a phase II clinical trial assessing the adjunctive benefit of CUR combined with paclitaxel for advanced breast cancer patients is ongoing (NCT03072992). 

In breast cancer, whether for prevention in high-risk obese population or as an active role in reducing radiation-induced dermatitis or enhancing paclitaxel effects/reducing toxicities, CUR is making its way through ongoing trials in breast cancer.

Curcumin is also being studied for its protective effects in anthracycline-induced cardiotoxicity and chemotherapy-induced nephrotoxicity. It also reduces cisplatin resistance by inhibiting FEN-1 (Flap endonuclease 1) expression in animal models. Whether it is combined with tyrosine kinase inhibitors in lung cancer, with gemcitabine in pancreatic cancer, pre-cancerous lesions in cervical cancer, or its role in sarcoma, CUR is expected to show its promising effects.

NCT03211104 is a placebo-controlled, double-blind, randomized trial investigating whether CUR influences the course of prostate cancer patients treated with on/off hormonal deprivation therapy. 

A larger phase III trial is investigating the risk-reducing potential of CUR in terms of recurrence-free survival for patients undergoing radical prostatectomy for an adenocarcinoma of the prostate (NCT02064673). In addition, another protocol is analyzing the role of CUR as a radio-sensitizing agent for prostate cancer patients, as assessed by tumor response. 

Furthermore, the role of CUR associated with standard treatments for cancer is being evaluated for non-small-cell cancer patients, receiving tyrosine kinase inhibitors for advanced disease (NCT02321293). Table 2 summarizes the studies described in this section.

The role of CUR in cancer treatment was addressed by numerous trials. Although apparently working at low plasma concentration, systemic bio-availability still represents an issue when dealing with CUR experiments in humans. Indeed, the optimization of CUR bio-availability through drug-delivery strategies provided more significant results, suggesting that in vitro and in vivo antitumoral activity can be replicated in the clinical setting by different pharmacological strategies. However, some of the results provided so far are no more than proof-of-concept studies, particularly for the combination with targeted agents, where only a few positive experiences are reported. Moreover, no phase III clinical trial provided results on the antitumor efficacy compared to the standard treatments, with efforts warranted in the definition of the disease most likely to benefit along with predictive biomarkers for better patient selection. For this, despite promising results and early signals of benefit, at present, no specific recommendation should be provided for CUR in the treatment of cancer, as data of safety are restricted to monotherapy and few combinations with selected agents, and data of activity are not mature enough. Eventually, the use of CUR to “adjuvate” the standard treatments should be discouraged unless new results will identify a precise setting of care as a pharmacological compound.

## 6. Conclusions

The majority of studies analyzing CUR in cancer showed potentially beneficial effects on side effects and eventually also additive or even synsergistic effects on the efficacy of classical anti-cancer drugs. However, data from recent clinical trials are not sufficient to implement CUR as standard anti-cancer treatment. Large randomized trials are urgently needed to investigate the real effect of CUR in hemato-oncology. However, the efficacy of CUR as a complementary and/or alternative medical approach is promising. In addition, CUR causes no significant side effects and is cheap and easily available, even though its poor bioavailabity and fast metabolism remain major obstacles. 

## Figures and Tables

**Table 1:** Interactions between curcumin (CUR) and different pathways.

Pathway
Reduction of radical oxygen species (ROS)
Inhibiton of the production of toxins via cytochrome P450
Inhibition of COX2 and NF-κB
Reduction of proinflammatory cytokines (i.e., IL-1, IL-2)
Induction of caspase pathway (i.e. caspase-3)
Downregulation of anti-apoptotic genes (i.e., Bcl-2)
Inhibition of growth promoters (i.e., EGFR)
Decrease of MMP
Anti-metastatic properties (i.e., inhibition of VEGF)
Inhibition of PI3K/Akt/mTor pathway
Inhibtion of HIFα pathway
Downregulation of the HGF/MET pathway
Downregulation of stemness markers (i.e., CD133, CD44)
Inhibtion of the Hedghog signaling pathway

**Table 2:** Currently ongoing clinical trials with curcumin.

NCT Number	Title	Cancer Type	Setting	Study Phase	Primary Objective
NCT02944578	Biomolecular Effects of Topical Curcumin in HSIL Cervical Neoplasia	Cervical pre-cancer lesions	Cancer prevention	2	Change in human papillomavirus (HPV)-related molecular target HPV E6/E7 messenger ribonucleic acid (mRNA) expression within HSIL lesions of the cervix
NCT02554344	Effect of Curcumin in Treatment of Squamous Cervical Intraepithelial Neoplasias (CINs)	Cervical pre-cancer lesions	Cancer prevention	1	Determine the safety and feasibility using curcumin in patients with CIN3 where toxicities will be graded according to the NCI Common Terminology Criteria for Adverse Events (CTCAE) Version 4.0.
NCT00927485	Use of Curcumin for Treatment of Intestinal Adenomas in Familial Adenomatous Polyposis (FAP)	Intestinal adenoma	Cancer prevention	na	To determine in a randomized, double-blinded, placebo-controlled study the tolerability and efficacy of curcumin to regress intestinal adenomas by measuring duodenal and colorectal/ileal polyp number, and polyp size in patients with FAP
NCT03769766	A Randomized, Double-Blind, Placebo-Controlled Trial of Curcumin to Prevent Progression of Biopsy Proven, Low-Risk Localized Prostate Cancer Patients Undergoing Active Surveillance	Prostate cancer	Cancer treatment/(neo) adjuvant treatment	3	The primary endpoint is the number of patients who have progressed at 2 years of follow-up defined as one of the following events: receipt of primary therapy for prostate cancer (e.g., prostatectomy, radiation, hormonal therapy) or pathologic progression (>4 cores involved, ≥50% of any core involved, or any Gleason score ≥7)
NCT01975363	Nanoemulsion Curcumin for Obesity, Inflammation, and Breast Cancer Prevention—A Pilot Trial	Breast cancer	Cancer prevention	na	To determine whether nanoemulsion curcumnin modulates pro-inflammatory biomarkers in plasma and breast adipose tissue
NCT02724202	A Pilot, Feasibility Study of Curcumin in Combination with 5-FU for Patients With 5-FU-Resistant Metastatic Colon Cancer	Colorectal cancer	Metastatic treatment	1	Determine the safety using curcumin in patients with metastatic colon cancer; where toxicities will be graded according to the NCI Common Terminology Criteria for Adverse Events (CTCAE) Version 4.0 (time frame: 12 weeks)
NCT02439385	First-Line Avastin/FOLFIRI in Combination with Curcumin-Containing Supplement in Colorectal Cancer Patients with Unresectable Metastasis	Colorectal cancer	Metastatic treatment	2	Progression-free survival
NCT03072992	Study of Efficacy of Curcumin in Combination with Chemotherapy in Patients with Advanced Breast Cancer: Randomized, Double-Blind, Placebo-Controlled Clinical Trial	Advanced cancer	Metastatic treatment	2	Objective response rate (time frame: 4 weeks after the completion of the treatment)
NCT03211104	Comparison of Duration of Treatment Interruption with or without Curcumin During the Off Treatment Periods in Patients with Prostate Cancer Undergoing Intermittent Androgen Deprivation Therapy: A Randomized, Double-Blind, Placebo-Controlled Tria	Prostate cancer	Metastatic treatment	na	Duration of treatment interruption with or without curcumin (time frame: up to 42 months)
NCT02064673	Randomized Trial of Adjuvant Curcumin after Prostatectomy	Prostate cancer	Adjuvant treatment	3	Serum prostate-specific antigen (time frame: 3 years) Recurrence-free survival defined as a total serum prostate specific antigen of <0.2 ng/mL.
NCT02321293	A Phase I Open-Label Prospective Cohort Trial of Curcumin Plus Tyrosine Kinase Inhibitors for Epidermal Growth Factor Receptor (EGFR)-Mutant Advanced Non-Small-Cell Lung Cancer	Non-small-cell lung cancer	Metastatic setting	1	Feasibility and safety
