# Erratum: Willenbacher, E., et al. Curcumin: New Insights into an Ancient Ingredient against Cancer. *Int. J. Mol. Sci.* 2019, *20*, 1808

**DOI:** 10.3390/ijms21165725

**Published:** 2020-08-10

**Authors:** Ella Willenbacher, Shah Zeb Khan, Sara Cecilia Altuna Mujica, Dario Trapani, Sadaqat Hussain, Dominik Wolf, Wolfgang Willenbacher, Gilbert Spizzo, Andreas Seeber

**Affiliations:** 1 Department of Internal Medicine V: Hematology and Oncology, Medical University of Innsbruck, 6020 Innsbruck, Austria; ella.willenbacher@i-med.ac.at (E.W.); dominik.wolf@i-med.ac.at (D.W.); wolfgang.willenbacher@tirol-kliniken.at (W.W.); gilbert.spizzo@i-med.ac.at (G.S.); 2 Department of Clinical Oncology, BINOR Cancer Hospital, Bannu 28100, Pakistan; skhanizhere0@gmail.com; 3 Department of Molecular Biology, Laboratorio Blau, Caracas 1071, Venezuela; altunamujica.md@gmail.com; 4 Department of Oncology and Hematology, University of Milan, IEO, European Institute of Oncology IRCCS, 20122 Milan, Italy; dario.trapani@ieo.it; 5 Medical Oncology Department, KAMC NGHA, Riyadh 14413, Saudi Arabia; oncologysh@gmail.com; 6 Oncotyrol, Center for Personalized Cancer Therapy, 6020 Innsbruck, Austria; 7 Oncologic Day Hospital, 39042 Bressanone, Italy

We would like to change the authors’ names and affiliations on Page 1 of paper [1] from:

^4^ Department of Oncology and Hematology, University of Milan, European Institute of Oncology, 20122 Milan, Italy; dario.trapani@ieo.it

to the correct version, as follows:

^4^ Department of Oncology and Hematology, University of Milan, IEO, European Institute of Oncology IRCCS, 20122 Milan, Italy; dario.trapani@ieo.it

We apologize for any inconvenience caused to the readers.
